# Epidemiology of Headache in Children and Adolescents—Another Type of Pandemia

**DOI:** 10.1007/s11916-020-00892-6

**Published:** 2020-08-25

**Authors:** Vera Nieswand, Matthias Richter, Gudrun Gossrau

**Affiliations:** 1 Headache Clinic, Pain Center, University Hospital and Faculty of Medicine Carl Gustav Carus, TU Dresden, Fetscherstr 74, 01307 Dresden, Germany; 2 Department of Pediatrics, University Hospital and Faculty of Medicine Carl Gustav Carus, TU Dresden, Dresden, Germany

**Keywords:** Pediatric headache, Headache epidemiology, Headache prevalence

## Abstract

**Purpose of Review:**

Headaches are not only responsible for restrictions in everyday life in adults. In children and adolescents, regular headaches lead also to reduced life quality and limitations in the social sphere, in school education, and in professional careers. Here, we provide an overview on the frequency of headache in children and adolescents with the aim of increasing awareness about this particular health issue.

**Recent Findings:**

Overall, headache prevalence in children and adolescents has been increasing in recent years. From various regions worldwide, data describing headache, its forms, and consequences are growing. In addition, factors frequently correlated with headache are repeatedly investigated and named: besides genetic factors, psychosocial and behavioral factors are linked to the prevalence of headache.

**Summary:**

Increasing evidence indicates that headache is underestimated as a common disorder in children and adolescents. Accordingly, too little emphasis is placed by society on its prevention and treatment. Thus, the extent of the social and health economic burden of frequent headaches in children and adolescents needs to be better illustrated, worldwide. Furthermore, the data collected in this review should support the efforts to improve outpatient therapy paths for young headache patients. Factors correlating with headache in pupils can draw our attention to unmet needs of these patients and allow physicians to derive important therapy contents from this data.

## Introduction

Population-based studies in the European Union (EU) have shown an overall 1-year prevalence of 79% for headache disorders. Headache disorders are ranked as the second most common cause of years of life spent with disability worldwide [1••]. In the EU, headache disorders cost national economies well over 100 billion euros annually [2].

Headaches are the most common type of pain in children and young people with effects on health-related quality of life (HrQoL), school attendance, social functioning [3]. In the global burden of disease study 2016, migraine was ranked first as the most disabling disease in the 15–49 age group [4••].

Until today, the consequences of frequent headaches in children are poorly characterized and early detection and treatment of young headache patients is too rare. Care structures for children and adolescents with regular headaches are not adapted to the needs. Due to lack of or late treatment, the impairments caused by headache change the lives of young people. However, several studies in the last years have addressed the question of increasing prevalence of pediatric and juvenile headache. Here, we summarize the available data to draw a picture of the landscape and upcoming health care topics.

### Search Strategy and Results

A PubMed search with the terms “epidemiology, prevalence, pediatric/children/adolescents and headache, migraine” has been carried out for publications with dates between 2015 and 2020. Original articles investigating the epidemiology of headache in children and adolescents have been included.

### Methods to Study Headache Prevalence in Children and Adolescents

Compared with studies in adults, the number of epidemiological studies on headache in children and adolescents is smaller and includes fewer countries worldwide. In addition, significant methodological differences between individual epidemiological studies limit their comparability. As concluded by an expert consensus group, epidemiological studies in children and adolescents should use a cross-sectional design with school-based samples and questionnaires which specify the investigated age group, collect detailed information, and require restricted time to be completed [5].

An example of such validated tools is the child and adolescent Headache-Attributed Restriction, Disability, and Social Handicap and Impaired Participation (HARDSHIP) structured questionnaire for mediated group self-administration by pupils at school. Most studies relied for data on socio-familial factors, headache characteristics, impact on daily activities, and medication on anonymous multiple-choice questionnaires [3, 6]. Comparison between data quality of structured interviews and prospective diary recordings revealed consistency for recording headache prevalence but inconsistency for reporting “free of headache”. In particular, 50% of adolescents who reported in structured interview no headache, were headache positive in prospective diaries [7•]. This is an indication that minor headaches play a minor role in the perception of many young people and that the number of adolescents with headache is likely underestimated in data derived from structured interviews.

To assess the impact of headache in children and adolescents, validated questionnaires such as the pediatric version of the migraine disability assessment score (PedMIDAS) has been found to be appropriate for epidemiological studies. Notably, PedMIDAS requires responses in numbers of days with headache in the preceding 3 months, but has also some limitations, such as its underestimation of the impact of migraine on non-school days and a reduced recall accuracy compared with a headache diary [8, 9]. KINDL® is another validated questionnaire which scores headache on a 4-point Likert scale and inquires in the preceding 4 weeks about health-related quality of life and the impact of diseases on it in patients < 18-year old (https://www.kindl.org/english/). The pediatric pain disability index is used primarily in chronic juvenile pain patients to assess the burden of pain in everyday life and might be less effective in assessing disability due to episodic headache [10]. Considering data deviation by recall in epidemiological headache studies, the question of whether a headache occurred yesterday can be very useful to reduce the recall bias.

### Recent Studies on Headache Prevalence

**Fig. 1:**
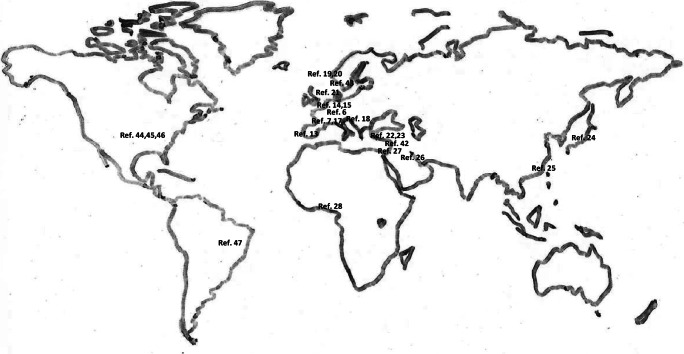
Presentation of headache prevalence studies worldwide

Headache in children and adolescents is a growing medical but also social task. This is supported on the one hand by the increasing use of medical facilities by children and adolescents with headaches in recent years, as shown in data from South Korea, Italy, and the USA [11–13]. On the other hand, various studies have investigated headache prevalence, mostly in school-based cross-sectional studies, with the conclusion that there is a high prevalence of headache in children and adolescents globally (Fig. 1, Table 1).

**Table 1:** Overview of juvenile headache prevalence studies

Country	Year	Age group	Participants	Prevalence overall	Girls	Boys	ICHD criteria				Methodology	HrQoL	Reference
							Migraine	TTH	UDH	Chronic			
Austria	2019	10–18 years	3386	75.7	82.1%	67.7%	ICHD-II: 24.2%	21.6%	26.1%	3%	Validated questionnaire	KIDSCREEN	Philipp et al. [3]
											1 year	HARDSHIP	
Spain	2019	12–18 years	1619	30.50%			ICHD-IIIβ: 11.3%			1.50%	Anonymous questionnaire	PedMidas	Torres-Ferrus et al. [14]
											Strengths and		
											Difficulties Questionnaire (SDQ)		
Germany	2019	6–19 years	2706	36.6% 1 day/month	≥ 2 headache days/month						anonymous questionnaires	item	Nieswand et al. [15]
				31.5% min. 2 days/month	34.2%	28.7%					3-month prevalence	“everyday life affection”	
Germany	2019	3–17 years	> 11,000		11–17 years:	11–17 years:					phone survey		Krause et al. [16]
					45.20%	28.70%					3-month prevalence		
					3–10 years:	3–10 years:							
					20%	16.40%							
Italy	2019	15 years	13,611	Mean of different regions	43–67%	20–37%					anonymous questionnaire		Centauri et al. [17]
				> 40%	Region-dependent								
Italy	2018	11–16 years	1950	65.90%							anonymous questionnaire		Foiadelli et al. [6]
				9.8% > 1/week							1 year		
				14.3% > 1/month									
				24.2% monthly									
				17.7% < once a month									
Crotia	2016	15–18 years	1876				ICHD-II: 12.8%	38.30%			anonymous questionnaire		Sedlic et al. [18]
							(17% girls, 8.1% boys)	(40.6% girls, 35.7% boys)			life time		
Norway	2015	12–18 years	493	88% 1 year			ICHD-II: 23% definite,	58% definite			face to face interview		Krogh et al. [19]
				38% point prevalence			13% probable				validated questionnaire		
											1 year		
Norway	2016	16–20 years	2594	79.40%			7.50%	19%			face to face interview		Jacobsena et al. [20•]
			Successive cohort	successive investigation			8.70%	21%			validated questionnaire		
			2373	77.50%							1 year		
Denmark	2018	11–15 years	31,102	8%							HBSC questionnaire [21]6 months		Holstein et al. [22]
				successive investigation									
				12.90%									
Turkey	2018	6–17 years	7088	68.80%			ICHD-IIIβ: 26.7%	12.90%	29.20%		validated questionnaire	HARDSHIP	Wöber et al. [23]
							(definite and probable)				1 year		
Turkey	2015	7–17 years	259,428	47.5% recurrent headache^a^			ICHD-II: 7.2	7.8			questionnaire		Poyrazoğlu et al. [24]
				26.4% primary headache							last 6 months		
Japan	2017	6–15 years	3285	49.40%			ICHD-IIIβ:				multiple choice questionnaire	item “interference	Goto et al. [25]
							Elementary school	Elementary school				with normal activities”	
							3.5%	5.4%				“concentration”	
							Junior high school	Junior high school					
							5.0%	11.20%					
Taiwan	2019	10–18 years	2309 5th–6th grade	63%							online survey		Miao et al. [26]
			2700 middle school										
			2013 high school										
China	2015	16–18 years	2849	30.30%							validated questionnaire	item “sleep disturbance”	Zhang et al. [27]
				≥ 1 day a week							previous 3 months	“academic pressure”	
Kuwait	2019	6–17 years	3423	19.4% primary headache	25.2%	13.8%	ICHD-II: 10.9%	6.20%		0.90%	validated questionnaire	HARDSHIP	Al-Hashel et al. [28]
											face-to face interviews		
											1 year		
Jordania	2019	16–18 years	754	66%			ICHD-II: 8.8%	19%	39%		questionnaire		Al Bashtawy et al. [29]
											recurrent		
Nigeria	2017	17–23 years	1500	23.70%			ICHD-II: 2.4%	12.50%	8.90%	3.50%	questionnaire		Sanya et al. [30]
											≥ 4 headache days last year		

*HrQoL* health-related quality of life, *HARDSHIP* Headache-Attributed Restriction, Disability, and Social Handicap and Impaired Participation, *HBSC* health behavior in school-aged children, *SDQ* strengths and difficulties questionnaire

^a^Last 6-month “recurrent headache”

Starting with Europe, a recent study from Austria found in a representative sample of 3386 pupils aged 10–18 years an overall 1-year headache prevalence of 75.7% (girls: 82.1%; boys: 67.7%) and increasing with age [3]. In addition, this study reported on headache types and differentiated a prevalence of 24.2% migraine, 21.6% tension type headache (TTH), 3.0% chronic headache on ≥ 15 days per month, and undifferentiated headache in 26.1%. Restrictions in daily life occurred in 42% of young people with headache and health-related quality of life was reduced in almost all children and adolescents with headache. Of the pupils with headache, 50% used medication for it—a prevalence which increased to 67% in those with chronic headache. Interestingly, pupils from single parent or patchwork families exhibited a higher probability of migraine (OR 1.5).

A current cross-sectional study from Spain investigated 1619 students of 12–18 years using an anonymous questionnaire [14]. The prevalence of recurrent headache was 30.5%, with 11.3% of probands showing migraine features; 32.9% of the subjects reported at least one headache episode per week, while 44.1% of them suffered from headache-related disabilities as measured by PedMIDAS. Headache was more frequent among girls (35.1%) than in boys (25.5%), in those who had a poorer sleep hygiene, less physical activity, skipped breakfast, smoked, or with frequent caffeine consumption. Chronic pain disorders, mental health problems, and allergies were also found to be significantly associated with headache.

A recently study of our group in Germany investigated headache prevalence in pupils aged 6 to 19 years using anonymous questionnaires and concluded that the majority of pupils suffer from headache at least once a month [15]. Of 2706 children and adolescents, 36.6% indicated headache once a month and 31.5% at least 2 headache days per month within the last 3 months. Headache correlated positively with increasing age, female gender, additional diseases, drug intake on a regular basis, analgesic ingestion for other diseases, caffeine consumption, and lack of sports. Headache and secondary school type showed a tight relationship: 67.2% of the pupils attending 8-year secondary schools (resulting in university entrance level II certificates as precondition for university studies) and 79.5% of those attending 6-year secondary schools (resulting in level I certificates, preparing for general professional training) reported headache. In addition, strong correlations were revealed between headache frequency and school absence and between headache intensity and headache frequency. Among pupils with headache ≥ 2 days a month, 48.1% indicated the intake of analgesics, with ibuprofen (49.1%) and paracetamol (32.8%) being the most frequently used. Moreover, 68.3% of the pupils in this group did not report a specific headache diagnosis.

Additional data from Germany collected through a phone shear survey in > 11,000 participants between 3 and 17 years indicated that in 11- to 17-year-old pupils, recurrent headaches were the leading cause of pain and affected almost every second girl and about every third boy [16]. The 3-month prevalence of recurrent headache, abdominal, and back pain increased in the last years especially in the age groups 7 to 10 years and 11 to 13 years. Teenagers assumed analgesics for repeated headaches almost twice as often compared with children of younger age.

A third German study investigated 1399 students aged 12–19 years with primary headache in the last 6 months for daily living activities, medical care utilization, and drug use. Overall impaired daily activities were predominant in adolescents with migraine. During the past year, only 12% of pupils with headache frequented a physician and approximately 30% were consuming analgesics for headache [31].

A nationwide Italian study using self-reported data from 13,611 15-year-old adolescents reported recurrent headache in almost 45% of the population [17]. In addition, > 30% reported recurrent backpain and 30% abdominal pain. Pain and analgesic consumption were higher in girls and adolescents. Adolescents with recurrent pain consumed analgesics more frequently for other health problems than for pain. Another recent survey in 1950 Italian adolescents of 11 to 16 years used anonymous multiple-choice questionnaires and reported an overall headache prevalence of 65.9%. Of those 9.8% reported headache > 1/week, 14.3% > 1/month, 24.2% monthly, and 17.7% < once a month. Analgesic intake was reported in 69.2% of adolescents, while only 20.6% had a physician’s prescription [6]. School was perceived as the major triggering factor in 67% and girls were more affected in daily life by headache than boys.

In Croatia, 1876 high school students completed a self-administered 36-item questionnaire [18]. Here, the prevalence of migraine was 12.8% (17% in girls and 8.1% in boys), of tension-type headache (TTH) 38.3% (40.6% in girls and 35.7% in boys). Students with migraine reported more analgesic intake, health care use, and smoking.

The prevalence and disability of headache among Norwegian adolescents have been investigated in a cross-sectional school-based study with 493 adolescents aged 12–18 years [19]. The overall 1-year headache prevalence was 88%, the point prevalence 38%. In detail, 1-year headache prevalence of definite migraine was 23%, of probable migraine 13% of TTH 58%, while in 9%, more than one headache type could be diagnosed. For Norway, additional data based on successive, cross-sectional, population-based studies report an increase in all types of recurrent headaches and a significant increase in TTH among adolescents [20•].

In Denmark, the correlation of economic inequality and frequent headache was investigated in 31,102 adolescents of 11 to 15 years between 1991 and 2014 [22]. Overall, 10.4% of adolescents reported frequent headache. A significant increase between 1991 and 2014, 8% versus 12.9%, respectively, was noticed in all economic classes. Lower socio-economic conditions correlated with higher prevalence of frequent headache (OR = 1.50, 95% CI: 1.34–1.67)—this phenomenon did not change over time.

A school-based questionnaire was administered to investigate 7088 pupils aged 6–17-year old in different regions of Turkey [23]. The overall 1-year prevalence of headache was estimated to be 68.8%. In detail, 29.2% reported undifferentiated headache, 26.7% migraine, and 12.9% TTH. Burden of headache was higher and quality of life lower in pupils with migraine and TTH. Another study in Turkish 7–17-year-old pupils rated the prevalence of recurrent headache to be 47.5%. Among these subjects, 21% suffered from primary recurrent headache [24].

A Japanese school-based questionnaire survey in 3285 students of 6–15 years revealed headache in 49.4%, migraine and TTH in 3.5% and 5.4% of elementary school students, and in 5.0% and 11.2% of junior high school students, respectively [25]. Migraine resulted in concentration disturbances and headache-related frustration more often than other headaches. Fifty percent of the students with headache-related disability did not receive medical treatment.

In 2014, a study among > 7000 pupils aged 10 to 18 years in Taiwan found 85.6% of children and adolescents having pain in the last year, and among these, 63% described headache [26].

In China, a study investigated 2849 high school students for recurrent pain. A total of 30.3% of the students described headache at least once a week in the previous 3 months. Sleep disturbances and academic pressure correlated to headache and other recurrent pain [27].

In Kuwait, a study investigated 3423 6–17-year-old children and adolescents using the HARDSHIP questionnaire and reported a 1-year prevalence of headache of 19.4% (migraine 10.9%, TTH 6.2%, chronic headache 0.9%) [28]. Primary headache disorder significantly increased in the 12–17-year-age group when compared with the 6–11-year-age group (25.8% vs. 10.4%; *p* < 0.001). One-year primary headache prevalence showed non-significant gender differences (10.1% in boys vs. 10.6% in girls; *p* value < 0.79). Yet, headache prevalence was significantly higher in females than in males in the 12–17-year-age group compared with the 6–11-year-age group (38.1% vs. 15.8%).

In 2017, a questionnaire study in Jordania among students aged 16–18 years reported an overall headache frequency of 2/3, among those 19.0% were classified as having TTH, 8.8% migraine, and 39.0% undifferentiated headache [29]. This study points to headache as one of the major public health problems among high school students and calls for new therapies.

In Nigeria of 1500 students aged 20.9 ± 3.1 years, 23.7% reported headache in the past year, and 3.5% chronic headache [30]. In detail, 12.5% had TTH, 2.4% migraine, 8.9% undifferentiated headache, and none of them had medical consultation for the headache.

In Brasil, 954 adolescents (14 to 19 years) have been studied for headache and use of computer and videogames [32]. Among them, 80.6% reported headache, including 17.9% with TTH, 19.3% with migraine, and 43.4% with undifferentiated headache. The excessive use of electronic devices was a major risk factor (OR = 1.21) for headache, and especially for migraine.

### Headache and Education

Regular headache leads to frequently missed school lessons, dropping out of school with poorer learning outcomes. Some patients have to repeat a school grade, or change the type of school toward a less qualified degree. As a consequence, the whole educational path can be negatively affected by headaches in childhood and adolescence.

In Israel, 262 children with headache between 5 and 18 years old have been investigated for learning disabilities [33]. Comparing children with migraine and those with both, migraine and TTH it turned out that in pupils with migraine and TTH the likelihood of learning disabilities was 2.7 times higher than in patients with migraine alone.

A Swedish investigation in 9th grade adolescents reported significantly lower school grades among those with headache or abdominal pain [34]. The relevant conclusion was that identification and treatment of pain symptoms may improve academic results.

A recent study investigated children aged 5 to 17 years in the US for the correlation between prevalent headache and school absenteeism. Frequency of severe headache in the previous 12 months was reported by caregivers in 6% of the investigated 57,272 children [35]. Interestingly, headache became more associated with school absenteeism over time.

Another North-American survey studied anonymously sleep, school performance, headache, and messaging habits in high school students [36•]. A correlation between hypersomnolence, chronic headaches and less sleep was shown, albeit not in students in lower school grades.

The relationship between school start time and headache frequency was investigated in a cross-sectional study of US high schoolers with migraine. A comparison between those who started school at 8:30 AM or later and those with earlier start time schools was carried out and reported no effect of school start time on headache days per month [37].

A Brazilian study in 195 elementary school students aged 10 to 15 years could clearly correlate severe headache and migraine with lower quality of life and lower academic performance [38].

## Conclusions

Headache disorders in children and adolescents affect frequently school and social activities and the work performance of the parents. Awareness of early diagnosis and preventive therapies must improve to prevent chronic headaches and its negative impact on performance at school and in the social context. The data summarized in this review highlight the need for improved management and prevention of headaches in children and adolescents.

### Excursion to the Practice I: Headache Diagnoses in Children and Adolescents

The primary task for pediatricians taking care of children and adolescents with headaches is to exclude a secondary headache. After a symptomatic headache has been clinically excluded with diagnostic methods such as cranial MRI and lumbar puncture, headache is often no longer addressed as a disorder of the young patient. Therefore, pediatric patients often suffer for months or years before receiving adequate treatment by a pediatrician, neurologist, or pain therapist specialized in headache.

At the beginning of a structured therapy, headaches should be classified as being either primary or secondary following the International Classification of Headache Disease 3rd version 2018 [39]. Red flags as fever, trauma, occipital headaches, suddenly raising headaches, or abnormalities in neurological examination point to underlying diseases such as meningitis, sinus venous thrombosis, intracerebral/subarachnoidal/epi- or subdural hemorrhage, brain tumor, stroke, intracranial hypertension or cerebrospinal fluid (CSF) leak, which all lead to secondary headaches. These secondary causes for headache have to be ruled out with appropriate diagnostic investigations [40].

Primary headaches in childhood and adolescence are most frequently TTH or migraine. In pediatric patients, migraine and TTH occur often together and the clinical presentation shows a mixture of both [41].

TTH is mostly bilateral, characterized by a dull, oppressive pain, up to medium intensity, ranging over a time of 30 min to 7 days, sometimes with photo/phonophobia, improvement by distraction, not worsening by physical activity and sometimes accompanied by increased cervical and pericranial musculature tone increase.

Migraine appears as uni- or bilateral, sharp, pulsating or dull, oppressive pain, up to high intensity, ranging over a time period of 60 min to 72 h (including sleep), mostly with photo/phonophobia, osmophobia, and vegetative syndromes such as nausea and vomiting, improvement by retreating in a dark room and sleep, worsening by physical activity, without any explainable other reason [42]. TTH and migraine are mostly episodic (less than 15 headache days a month), rarely chronic with more than 15 headache days and in chronic migraine at least 8 migraine days per month. Migraine can appear without or with aura symptoms, in the latter case complicated by neurological findings including hypoaesthesia with a migrating aspect (e.g., starting at the finger, involving elbow and tongue) or positive scotoma.

Trigeminal autonomous headaches are very rare in children and adolescents, while other primary headaches such as new daily persistent headache (persistent headache which started at a defined time point with a permanent persistent headache often with high migraine-like intensity) have so far been little studied [43•, 44].

At the beginning of treatment, proper classification of the headache is of critical importance. After secondary headaches have been excluded, patient and family education for the individual headache is the basis for further therapeutic work. In particular, the physician should explain how to treat the headache and what the child or adolescent and the family can do themselves.

### Excursion to the Practice II: Headache Management in Children and Adolescents

Therapy for headaches consists out of three pillars: activation, relaxation, and medication. Dependent on the individual headache diagnosis and disease stage, the therapeutic approaches are different.

#### Acute Medication

The recommended medications are ibuprofen or acetaminophen. These should be used in a body weigh adjusted manner (10 mg/kg/BW for ibuprofen, 15 mg/kg/BW for acetaminophen) early during migraine attacks (hit hard and early), other NSAIDs (e.g., naproxen) are alternatives. Adolescent migraine patients can profit from combined medications such as acetaminophen, acetyl salicylic acid, and caffeine during headache episode.

Triptanes can be prescribed from the age of 12-year old, although specialists may use triptans in younger patients if needed. The starting triptane is 10 mg sumatriptan as nasal application < 30 kg BW, and  20 mg >30 kg BW. Alternatively, zolmitriptan is available as nasal spray [45].

The take home message for pediatric patients is to follow the “3 to 10” rule. This means, do not use acute headache medication more than 3 successive days and not more than 10 days per month. All acute medications can lead to analgesic induced headache, if ingested for more than 10 days a month. If nausea and vomiting are frequent, nasal, sublingual, rectal, or subcutaneous application forms of triptans are indicated and in addition the use of antiemetics, i.e., dimenhydrinate or domperidone should be considered.

#### Prophylactic Medication

None of the prophylactics used in adults have shown reliable effects in high quality RCTs in children and adolescents, likely due to placebo effects in young patients. Nevertheless, prophylactic drugs in young migraine patients such as flunarizine, metoprolol, amitriptyline, topiramate should be considered, if all non-medication therapies do not decrease the migraine frequency and individual disease load is high. Importantly, when using these drugs information and education of the patient and of the parents is needed as well as a close clinical follow-up [46, 47]. Magnesium as nutritional supplement represents an alternative prophylactic, which should be used 10 mg/kg/BW per day. A side effect of this supplementation can be bowel irritation, in this case a dose reduction is recommended.

Activation as headache prophylaxis includes regular sport twice a week, best in a group of peers, with the aim of enjoying movements in a non-competitive setting.

Relaxation includes regular day structure and daily break of 10 min, which should be accompanied by relaxation techniques such as “safe area imagination” or breathing techniques. It is important though to comfort the child or adolescent, such that they do not feel compelled to reach high goals, and in so doing mitigate the risk of further migraine episodes [48]. Besides improvement of headache scores, studies show that cognitive behavioral therapy can lead to functional changes in brain function being beneficial for migraine therapy [49•]. These techniques should be accompanied by teaching of the parents on how to handle the headache situation [50].
